# Inequity in the provision of and access to palliative care for cancer patients. Results from the Italian survey of the dying of cancer (ISDOC)

**DOI:** 10.1186/1471-2458-7-66

**Published:** 2007-04-27

**Authors:** Monica Beccaro, Massimo Costantini, Domenico Franco Merlo, the ISDOC Study Group

**Affiliations:** 1 Unit of Clinical Epidemiology, National Cancer Institute, Genova, Italy; 2 Unit of Epidemiology and Biostatistics, National Cancer Institute, Genova, Italy

## Abstract

**Background:**

The palliative services and programs have been developed with different intensity and modalities in all countries. Several studies have reported that a geographic variation in the availability and provision of palliative care services between and within countries exists, and that a number of vulnerable groups are excluded from these services. This survey estimates the distribution of places of care for Italian cancer patients during the last three months of their lives, the proportion receiving palliative care support at home and in hospital, and the factors associated with the referral to palliative care services.

**Methods:**

This is a mortality follow-back survey of 2,000 cancer deaths identified with a 2-stage probability sample, representative of the whole country. Information on patients' experience was gathered from the non-professional caregiver through an interview, using an adapted version of the VOICES questionnaire. A section of the interview concerned the places of care and the palliative care services provided to patients. Multivariate logistic regression analyses were conducted to identify the determinants of palliative care service use.

**Results:**

Valid interviews were obtained for 67% of the identified caregivers (n = 1,271). Most Italian cancer patients were cared for at home (91%) or in hospital (63%), but with substantial differences within the country. Only 14% of Italian cancer patients cared for at home against 20% of those admitted to hospital, received palliative care support. The principal determinants identified for receiving these service were: an extended interval between diagnosis and death (P = 0.01) and the caregiver's high educational level (P = 0.01) for patients at home; the low patient's age (P < 0.01) and the caregiver's high educational level (P = 0.01) for patients in hospital.

**Conclusion:**

In Italy palliative care services are not equally available across the country. Moreover, access to the palliative care services is strongly associated with socio demographic characteristics of the patients and their caregivers. Italian Policy-makers need to equalise palliative care provision and access across the country to meet the needs of all cancer patients.

## Background

The provision of palliative care services and programs for terminal ill patients has become an important public health issue during recent decades. The worldwide development of hospices and the growing number of hospital and domiciliary palliative care teams (PCTs) differ between countries, reflecting the heterogeneity of the health care systems, patient's needs, and cultures [1-3]. Recent studies show that a remarkable geographic variation exists in the availability and provision of hospice and other palliative care services [4-6] also within the countries. Two literature reviews show that access and referral to specialist palliative care services are unequally distributed among cancer patients [4,7]. Cancer patients are less likely to receive palliative care if they are elderly, have no informal carer support, a low socio-economic status, poor functioning and a high nursing care requirement, a short term survival and a diagnosis of haematological, gastrointestinal, or central nervous system (CNS) tumours [4,7-14].

In all Western countries a very high proportion of terminally ill cancer patients are cared for and die in acute care hospitals where often PCTs are lacking [15-19]. As far as we know, previous studies have not identified the factors associated with the utilization of hospital-based palliative care support.

Published research on this topic, is based on geographically restricted populations that are often limited to specific palliative care programs existing in that region. [4,7-14,20] No population-based study, estimating at a national level, the spatial distribution, availability and accessibility of palliative care services for all cancer patients is available.

The Italian Survey of the Dying Of Cancer (ISDOC) is a mortality follow-back survey conducted in a stratified random sample of Italian deceased from cancer between March 2002 and June 2003. Information regarding the advanced and terminal phase of cancer was obtained from the caregivers, interviewed after the patient's death by using the View of Informal Carers – Evaluation of Services (VOICES) questionnaire [21].

ISDOC's aim is to use the information gathered during the interviews to provide national estimates of the end of life care experiences of terminal cancer patients in Italy.

This paper reports the results from specific sections of the interview focused on the places of care and the palliative care services provided to cancer patients during the last three months of life. Specifically, the analysis is aimed at:

• describing the places of care (home, hospital, nursing home and hospice) of cancer patients in Italy during the last three months of life;

• evaluating the validity of information concerning the referral for domiciliary PCTs, as reported by the caregivers during the interviews;

• estimating the proportion of Italian cancer patients who received palliative care support at home or in hospital;

• analyzing the socio-demographic factors associated with the referral of cancer patients in Italy for domiciliary PCTs, for hospital PCTs or pain specialists during hospital admissions.

## Methods

### The Italian Survey of the Dying Of Cancer (ISDOC)

A 2-stage probability sample was used to estimate end-of-life outcomes of approximately 160,000 annual cancer deaths in Italy. In the 1^st ^sampling stage, 30 out of 197 existing Local Health Districts (LHD) were randomly selected, following stratification by four Italian geographical areas. In the 2^nd ^stage, a fixed proportion of adult cancer deaths (aged 18 years or more) were drawn from each LHD, contributing to a sample of 2,000 death certificates of deceased for cancer between March 2002 and June 2003 [21].

For each case included in the survey, the non-professional caregiver, who was defined as the closest and the best informed person about the patient's last three months of life, was identified. A letter was sent to all identified caregivers to inform them of the study aims and obtain formal consent to be interviewed. Three to 10 days later, a trained interviewer contacted the identified caregiver to discuss the interview in detail. Information regarding the methodology of the survey has been published in a previous article [21].

The non-professional caregiver was identified for 92.1% of deceased patients (n = 1,843). For 57cases, with no recognized non-professional support (2.9%), the professional caregiver was identified.

An interview was conducted with 1,289 (67.8%) of the 1,900 identified caregivers at an average of 234 days after the patients' death (range 103–374). Of the remaining 611 non-interviewed caregivers, 161 (8.5%) could not be located, 383 (20.1%) refused to be interviewed, 38 (2.0%) were too ill to participate, and 7 (0.4%) were deceased. Twenty-two interviews (1.1%) were not carried out due to staff errors during planning. Finally excluded from all the statistical analyses were: 6 patients, whose death was thought not to be due to cancer, 12 who did not reach the terminal phase of disease (they died during the diagnostic phase, active treatments or diagnosis was post-mortem) [21]. The study design was approved by the Ethical Committee of National Cancer Institute of Genoa, and, according to the Italian law on use and processing of health data, a notification of the study design and procedures was sent to the Italian Data Protection Commission.

### Data collection

The interviewer met the caregiver, usually in his/her home, where she/he conducted a semi-structured interview using an adapted version of the VOICES questionnaire [22]. A specific section of the interview concerned information regarding care settings and the palliative care services delivered to the cancer patients. The questions asked to the caregiver are reported below.

 During the last three months of life, did the patient spend any time at home, in a nursing home, in hospital or in a hospice?

**Home**: While the patient was at home, was she/he referred for domiciliary PCTs? If YES, please specify the date of the first home visit.

**Hospital: **While the patient was in hospital, was a pain specialist involved in her/his care?

The information regarding admission to hospital in the last three months of life concerned:

• If the patients died in hospital, the last admission to hospital (for the admissions > 24 hours);

• If the patients died elsewhere, the longer admission to hospital.

### Validity of the information collected during the interview regarding domiciliary PCTs

To verify the validity of the information reported by the caregivers during the interview concerning the referral of the patients for domiciliary PCTs, we contacted, at the end of the study, all domiciliary PCTs active in the Local Health Districts (LHD) sampled for this study. The identified sample of cancer deaths was cross-checked with the database of domiciliary PCTs, in order to obtain a valid list of patients who were followed or not, by the domiciliary PCTs (and the date of the first home visit).

All statistical analyses were based on socio demographic characteristics of patients and their caregiver collected during the interviews. The referral of patients for PCTs during the time they spent at home was obtained from the case sheets. The domiciliary PCTs case sheets, containing comprehensive and detailed information regarding the referral and the date of the first home visit has been considered to be the "gold standard",

### Statistical methods

All analyses were performed using SUDAAN version 9.0.1 (Research triangle Institute, Research Triangle Park, NC). This software, for the point and SE statistics estimation, takes into account four characteristics of complex survey data: the unequal probability selection of observations, the clustering of observations, stratification, and non-response. Sampling weights were introduced to obtain unbiased weighted point and standard errors (SE) estimates of the target population. Specifically, weights were introduced to adjust for the different probability to be selected in each of the four strata (the LHD were sampled disproportionately in each stratum), and to adjust for the different proportion of valid information obtained in each of the 30 LHD selected. The number of cancer deaths in different subgroups, have been estimated, using 1998 mortality data.

The level of agreement between information reported by the caregiver during the interview and data collected from the domiciliary PCTs case sheets, was examined using kappa statistics. Moreover, sensitivity and specificity of the information collected during the interviews were evaluated assuming that the information reported in the case sheets was the "gold standard".

The differences in the distribution of categorical variables (i.e., gender and marital status, place of residence, primary tumor, and caregiver relationship) were tested by means of chi-square test for heterogeneity. For ordinal variables, such as age at death, education, months since diagnosis, number of cohabitants, age, and education of the caregivers, the differences in their distribution were tested by means of chi-square test for trend.

Two multivariate logistic regression models were performed to examine the associations between patients' and caregivers' characteristics (the independent variables) and the referral of patients for PCTs at home (first model) and to a pain specialist during hospital admission (second model). Patients' and caregivers' characteristics (i.e. age at death, gender, education, marital status, place of residence, primary tumour, months since diagnosis and number of cohabitants for patients, and caregiver's relationship, age, gender and education) were all included in the logistic regression models. Variables with P > 0.10 were removed from each model by means of a step-down procedure to obtain the final models. For the variables included in the final logistic models, the strength of the association was estimated in terms of odds ratio (OR), the ratio of the odds to be referred to PCTs at home (first model) or to a pain specialist (second model) among patients in a given category to the corresponding odds in a reference category. In each model, the chi-square test statistics for heterogeneity and for trend were used to test the associations between the dependent variable and the independent categorical and ordinal variables, respectively.

To compare the availability of the domiciliary and hospice PCTs in the 30 LHD examined in the ISDOC study in 2002–2003, with current availability (1^st ^June 2006) all local palliative care referents were contacted and the Italian Society of Palliative Care (SICP) [23] database consulted.

## Results

### Places of care (Table 1)

The estimated proportion of Italian cancer patients who spent all or part of the last three months of life at home was 90.8% (95% CI: 88.0 – 93.0). 63.4% (95% CI: 59.0 – 67.7) of the patients were admitted to hospital during this period. A small but non-negligible proportion of patients (8.3%; 95% CI: 6.2 – 11.0) spent this time in a nursing home and only 0.7% (95% CI: 0.3 – 1.8) in a hospice.

The highest proportion of patients cared for at home was in the Southern regions of Italy (97.8%) as compared to the other regions. Conversely, patients were more frequently admitted to hospital in the Northern regions (73.5% in the North East and 66.4% in the North West) as compared to Southern regions (51.2%). The admission to nursing homes was negligible in the South (0.9%) compared to the Central and Northern regions (from 6.5% in Central Italy to 13.5% in the North East).

About one third (31.3%) of patients spent the whole of the last period of their life at home, 4.5% in nursing homes, and 2.9% in hospital. The most frequently reported combination was home and hospital, that accounted for 56.9% of the sample. Statistically significant differences among the 4 geographical areas (P < 0.001) were observed. Particularly striking was the proportion of patients cared for at home for the whole period in the South of Italy (47.9%), as compared to the others geographical areas, where the proportion of home cared for ranged between 17.0% in the North East and 32.3% in the Central regions.

**Table 1:** places of care of Italian cancer patients in the last three months of life.

	North West	North East	Center	South	Italy
Survey sample	604	209	241	217	1,271
Italian cancer deaths ^3^	47,988	32,954	35,772	38,732	155,446
		
**Place of care **^1^	% ^2^	% ^2^	% ^2^	% ^2^	% ^2^
Home	89.1	83.0	92.7	97.8	90.8
Hospital	66.4	73.5	63.5	51.2	63.4
Nursing home	11.9	13.5	6.5	0.9	8.3
Hospice	0.5	1.4	1.1	-	0.7
		
**Places of care (in detail)**					
Only home	26.9	17.0	32.3	47.9	31.3
Only nursing home	5.5	7.7	3.9	0.9	4.5
Only hospital	2.7	6.5	1.7	1.3	2.9
Home and hospital	58.0	61.7	58.5	49.9	56.9
Nursing home and hospital	2.7	1.9	1.4	-	1.6
Others	4.1	5.2	2.3	-	2.9
Total	100	100	100	100	100
	P < 0.001 (test for heterogeneity)				

^1 ^The sum is greater than 100 because a number of patients was in different places.

^2 ^All percentages are weighted.

^3 ^The number of cancer deaths have been estimated, using 1998 mortality data.

**Table 2:** validity of information regarding the referral for domiciliary PCTs reported by the caregivers.

		Domiciliary PCTs case sheets ^1^		
		
Caregivers^1^		YES	NO	TOTAL
	YES	167 (14.8)	25 (2.2)	192 (17.0)
	NO	19 (1.7)	918 (81.3)	937 (83.0)
		
	TOTAL	186 (16.5)	943 (83.5)	1,129 (100)

^1 ^For each cell number and (total percentages) are reported.

PCTs= Palliative Care Teams

**Table 3:** determinants of referral for domiciliary PCTs by selected characteristics.

	1998 Italian cancer deaths ^3^	Referred to domiciliary PCTs	
		
	No.	% ^2^	(95%CI)
AGE AT DEATH (years)			
18–54	10,296	13.5	(6.7 – 25.4)
55–64	19,451	16.4	(8.8 – 28.6)
65–74	44,336	14.9	(8.3 – 25.4)
75–84	55,093	15.0	(8.8 – 24.4)
85+	26,270	5.8	(2.6 – 12.6)
		P = 0.01	
GENDER			
Males	89,265	13.9	(8.5 – 21.9)
Females	66,181	12.9	(7.2 – 22.2)
		P = 0.66	
EDUCATION (years)			
≤ 5	103,804	12.0	(7.0 – 19.8)
6–9	26,699	15.8	(9.3 – 25.6)
10+	24,628	17.6	(9.5 – 30.3)
		P = 0.08	
MARITAL STATUS			
Single ^1^	61,103	9.8	(5.4 – 17.1)
Married	93,220	16.1	(9.8 – 25.4)
		P = 0.01	
PLACE OF RESIDENCE			
North West	47,988	18.1	(8.7 – 33.9)
North East	32,954	17.6	(7.5 – 36.1)
Center	35,772	15.6	(4.7 – 40.9)
South and islands	38,732	2.3	(0.6 – 8.2)
		P = 0.04	
PRIMARY TUMOR			
Head and neck	3,455	16.6	(6.5 – 36.3)
Digestive system	56,114	12.4	(7.4 – 20.1)
Respiratory system	33,510	13.7	(7.1 – 24.9)
Breast	15,269	15.4	(7.8 – 28.1)
Genitourinary system	22,766	19.4	(11.5 – 31.0)
Haematological	11,487	5.9	(2.7 – 12.5)
Others and unspecified	12,845	10.9	(5.6 – 19.9)
		P = 0.19	
MONTHS SINCE DIAGNOSIS			
1–3	28,657	9.6	(5.1 – 17.5)
4–6	20,397	9.4	(4.3 – 19.2)
7–12	27,612	15.1	(7.9 – 26.5)
13–36	41,260	15.1	(9.1 – 24.1)
> 36	31,737	17.9	(10.2 – 29.6)
		P < 0.01	
NUMBER OF COHABITANTS			
None	19,098	8.5	(3.4 – 19.6)
1	64,791	16.0	(10.2 – 24.0)
2	35,634	15.0	(7.7 – 26.8)
3+	35,923	10.3	(4.7 – 21.2)
		P = 0.69	
CAREGIVER (relationship)			
Spouse – partner	48,181	15.9	(9.5 – 25.3)
Child	71,696	14.1	(8.3 – 23.0)
Others	35,568	9.0	(4.5 – 17.1)
		P = 0.03	
CAREGIVER'S AGE			
18–44	42,025	13.0	(7.3 – 22.1)
45–54	35,486	13.8	(7.7 – 23.5)
55–64	32,075	14.0	(7.2 – 25.5)
65–74	26,364	12.6	(7.5 – 20.5)
75+	10,245	11.9	(6.0 – 22.2)
		P = 0.85	
CAREGIVER'S GENDER			
Males	47,477	11.6	(6.7 – 19.3)
Females	107,969	14.3	(8.6 – 22.9)
		P = 0.13	
CAREGIVER'S EDUCATION			
≤ 5		10.1	(5.4 – 18.0)
6–9	30,583	11.9	(6.2 – 21.8)
10+	69,702	15.6	(9.8 – 23.9)
		P = 0.02	
TOTAL	155,446	13.5	(8.1 – 21.5)

^1 ^Including widowed, separated and divorced.

^2 ^All percentages are weighted.

^3 ^The number of cancer deaths have been estimated from the study sample, using 1998 Italian mortality data.

**Table 4:** determinants of involvement of pain specialist in hospital by selected characteristics.

	1998 Italian cancer deaths ^1^	Followed in hospital by a specialist in pain	
		
	N.	% ^3^	(95%CI)
AGE AT DEATH (years)			
18–54	7,599	41.5	(24.9 – 60.3)
55–64	14,449	29.2	(20.4 – 40.0)
65–74	29,585	16.0	(11.5 – 22.0)
75–84	33,982	17.0	(11.4 – 24.2)
85+	12,999	11.4	(6.2 – 20.2)
		P < 0.01	
GENDER			
Males	60,469	19.1	(13.9 – 25.6)
Females	38,144	20.5	(14.6 – 28.0)
		P = 0.74	
EDUCATION (years)			
≤ 5	65,804	16.7	(12.0 – 22.8)
6–9	15,114	22.2	(15.3 – 30.9)
10+	17,696	28.2	(19.5 – 39.0)
		P = 0.01	
MARITAL STATUS			
Single ^2^	34,554	20.0	(14.2 – 27.6)
Married	63,130	18.9	(15.0 – 23.6)
		P = 0.72	
PLACE OF RESIDENCE			
North West	31,869	24.6	(19.8 – 30.1)
North East	24,208	13.3	(7.9 – 21.6)
Center	22,716	22.3	(13.3 – 25.0)
South and islands	19,821	16.3	(6.6 – 34.7)
		P = 0.12	
PRIMARY TUMOR			
Head and neck	1,880	14.8	(5.8 – 32.9)
Digestive system	40,314	19.8	(14.8 – 25.9)
Respiratory system	22,144	21.9	(14.2 – 32.2)
Breast	7,205	13.4	(6.3 – 26.1)
Genitourinary system	12,311	23.3	(14.2 – 35.8)
Haematological	7,387	15.7	(5.9 – 35.7)
Others and unspecified	7,371	16.9	(9.5 – 28.4)
		P = 0.54	
MONTHS SINCE DIAGNOSIS			
1–3	25,833	17.4	(11.9 – 24.8)
4–6	12,041	19.2	(11.8 – 29.8)
7–12	16,624	18.8	(10.9 – 30.4)
13–36	23,213	19.6	(13.4 – 27.8)
> 36	18,304	24.8	(16.8 – 35.0)
		P = 0.21	
ADMISSION'S WARD			
Surgery	20,547	21.1	(14.9 – 29.0)
Medicine	55,237	19.1	(14.0 – 25.6)
Oncology-Hematology	13,815	21.9	(13.5 – 33.5)
Other	9,014	15.6	(7.9 – 28.7)
		P = 0.81	
NUMBER OF COHABITANTS			
None	7,178	12.8	(6.2 – 24.7)
1	37,988	18.9	(14.0 – 25.1)
2	22,412	20.9	(12.5 – 32.8)
3+	20,797	18.5	(11.7 – 28.1)
		P = 0.58	
CAREGIVER (relationship)			
Spouse – partner	28,795	19.3	(13.0 – 27.7)
Child	44,759	17.0	(12.5 – 22.8)
Others	14,820	23.5	(15.0 – 34.2)
		P = 0.45	
CAREGIVER'S AGE			
18–44	28,797	22.6	(16.9 – 29.4)
45–54	23,331	20.4	(14.2 – 28.4)
55–64	20,080	20.5	(12.9 – 31.0)
65–74	16,040	13.6	(7.3 – 24.1)
75+	6,127	19.2	(8.5 – 37.9)
		P = 0.17	
CAREGIVER'S GENDER			
Males	26,679	22.8	(17.4 – 29.2)
Females	61,695	17.1	(12.5 – 22.9)
		P = 0.13	
CAREGIVER'S EDUCATION			
≤ 5	23,879	12.3	(7.9 – 18.4)
6–9	19,490	16.2	(8.7 – 28.4)
10+	41,114	24.5	(18.5 – 31.8)
		P < 0.01	
TOTAL	98,614	19.6	(15.5 – 24.5)

^1 ^Subgroup of cancer deaths who spent part of their last three months of life in hospital. The number of cancer deaths have been estimated from the study sample, using 1998 Italian mortality data.

^2 ^Including widowed, separated and divorced.

^3 ^All percentages are weighted.

### Validity of information collected during the interviews about domiciliary palliative care teams (Table 2)

Agreement between the two sources of information (those reported in the case sheets and those collected during the interview with the caregivers) about the referral for domiciliary PCTs was high (kappa = 0.86). The overall prevalence of referral from the two sources was similar (16.5% and 17.0%, respectively), and the misclassification rate minimal. Assuming the domiciliary PCTs case sheets as "gold standard", the sensitivity and specificity of the interviews with the caregivers as a source of this information was 89.8% (95% CI 85–94) and 97.3% (95% CI 96–98), respectively.

Information regarding the date of referral of 183/186 (98.4%) patients was collected through the case sheets. The average time in care was 100.2 days (SD = 223.6) (median 46). Conversely, information regarding the date of referral of 126/167 (75.5%) patients was collected through the caregivers' interview. According to the caregivers, the average time in care was 92.3 days (SD = 136.5) (median 56).

The proportion of patients who died within 7 days of referral was 9.3% in the case sheets and 9.5% reported by the caregivers. The proportions of patients that lived longer than 180 days was 9.3% (case sheets) and 10.3% (caregivers).

### Referral to domiciliary PCTs (Table 3)

Overall, the estimated proportion of Italian cancer patient referred for domiciliary PCTs was 13.5% (95% CI: 8.1–21.5). These patients were less frequently elderly (patients aged 85 years or more were 5.8%), single (9.8%), and lived in the South of Italy (2.3%). The proportion of patients with haematological tumours referred to domiciliary PCTs was very low (5.9%) compared to all other type of tumours. An increasing linear trend was observed with the period since diagnosis (P < 0.01), the caregiver's educational level (P = 0.02), and, although with a P = 0.08, with the patient's educational level. Patients with a spouse-partner or child as a caregiver had a greater likelihood to be referred for domiciliary PCTs (P = 0.03). There were no significant differences in the referral for domiciliary PCTs by patient's gender (P = 0.66), number of cohabitants (P = 0.69), caregiver 's gender (P = 0.13) or age (P = 0.85).

### Involvement of a pain specialist during hospital admissions (Table 4)

Overall, the estimated proportion of Italian cancer patient followed by a pain specialist during hospital admissions was 19.6% (95% CI 15.5–24.5).

A decreasing linear trend was observed with patient's age (P < 0.01) and a positive association with patient's and caregiver's educational level (P = 0.01 and P < 0.01, respectively). There were no significant differences by patient's gender (P = 0.74), marital status (P = 0.72), place of residence (P = 0.12), type of tumour (P = 0.54), months since diagnosis (P = 0.21), number of cohabitants (P = 0.58), type of ward (P = 0.81), relationship (P = 0.45), caregiver's gender (P = 0.13), and age (P = 0.17).

Because of the negligible proportion (8.3%) (Table 1) of patients cared for in a nursing home no analysis was carried out for this group. Overall, 12.3% (95% CI: 6.3 – 22.9) of these patients was followed by a pain specialist whilst in a nursing home.

### Factors associated with the referral for domiciliary PCTs and with the involvement of a pain specialist in hospital – Multivariate analyses (Table 5)

**Table 5:** Multivariate logistic regression analyses of determinants of referral.

	Referred for domiciliary PCTs		Referred for specialist in pain in hospital	
		
	OR	(95% CI) ^1^	OR	(95% CI) ^1^
**The patient**				
Age at death (years)				
18–54	ref.		ref.	
55–64	1.04	(0.40 – 2.68)	0.67	(0.27 – 1.63)
65–74	0.98	(0.38 – 2.54)	0.34	(0.14 – 0.83)
75–84	0.98	(0.37 – 2.59)	0.32	(0.13 – 0.74)
85 +	0.43	(0.16 – 1.14)	0.15	(0.06 – 0.41)
	(P = 0.10)		(P < 0.01)	
Place of residence				
North West	ref.		ref.	
North East	1.08	(0.27 – 4.30)	0.39	(0.20 – 0.75)
Center	0.80	(0.16 – 3.90)	0.75	(0.35 – 1.61)
South and islands	0.13	(0.02 – 0.69)	0.54	(0.20 – 1.44)
	(P = 0.09)		(P = 0.04)	
Months since diagnosis				
1–3	ref.		ref.	
4–6	0.60	(0.20 – 1.81)	1.42	(0.68 – 3.00)
7–12	1.06	(0.40 – 2.78)	0.92	(0.40 – 2.10)
13–36	1.44	(0.63 – 3.29)	1.07	(0.52 – 2.19)
> 36	1.75	(0.86 – 3.57)	1.47	(0.76 – 2.83)
	(P = 0.01)		(P = 0.44)	
**The caregiver**				
Relationship				
Spouse	ref.		ref.	
Child	0.72	(0.51 – 1.01)	0.85	(0.45 – 1.61)
Others	0.49	(0.28 – 0.85)	1.20	(0.52 – 2.80)
	(P = 0.03)		(P = 0.47)	
Gender				
Males	ref.		ref.	
Females	1.40	(0.93 – 2.11)	0.88	(0.54 – 1.44)
	(P = 0.06)		(P = 0.64)	
Education (years)				
≤ 5	ref.		ref.	
6–9	1.44	(0.82 – 2.51)	1.31	(0.63 – 2.74)
10+	2.00	(1.20 – 3.35)	2.36	(1.25 – 4.46)
	(P = 0.01)		(P = 0.01)	

^1 ^OR (95% CI) estimated by multivariate logistic regression with all patient's and caregiver's covariates included in the model. Only covariates with a P-value < 0.10 for at least one of the two models are reported.

Two multivariate logistic models were fitted to the same data, using as dependent variables the referral for PCTs at home (first model) and to a pain specialist in hospital (second model).

The probability of being referred for a domiciliary PCTs significantly increased with an extended time interval from diagnosis to death (P = 0.01) and caregivers with a higher educational level (P = 0.01). For caregivers other than the spouse, the probability of referral was significantly lower (P = 0.03). Referral was less likely for patients living in the South (OR = 0.13; 95%CI = 0.02–0.69), as compared to patients living in the other parts of Italy.

The probability of being followed by a pain specialist whilst in hospital significantly decreased with patient's increased age at death (P < 0.01), and it was minimal for patients aged 85 years or more (OR = 0.15; 95% CI 0.06 – 0.41). This probability increased significantly with the caregiver's with a higher educational level (P = 0.01). The involvement of pain specialist was more likely for patients living in the North East and in the South as compared to patients living in the other geographical areas (P = 0.04).

### Trend in domiciliary PCTs and hospices availability (Table 6)

**Table 6:** domiciliary PCTs and hospices in Italy in the 30 LHDs sampled in ISDOC study.

	2002–2003 (ISDOC)					at June 2006				
		
	Italy (n = 30)	NW (n = 9)	NE (n = 6)	Center (n = 7)	South (n = 8)	Italy (n = 30)	NW (n = 9)	NE (n = 6)	Center (n = 7)	South (n = 8)
DOMICILIARY PCTs										
Total number	23	13	4	4	2	24	13	4	4	3
LHD with 1 or more PCTs	16	8	3	3	2	17	8	3	3	3
INPATIENT HOSPICE										
Total number	3	1	1	1	-	12	6	4	2	-
Total beds	24	10	6	8	-	128	79	31	18	-
LHD with 1 or more hospice	3	1	1	1	-	7	3	3	1	-

PCTs = Palliative Care Teams

LHD= Local Health District

Table 6 shows the trends of the development of domiciliary PCTs and hospices in the 30 sampled LHD in the last 4 years. The number of domiciliary PCTs remained almost the same in all geographical areas during this time window. Overall, the number of hospices increased 4 fold (from 3 to 12) and the total beds available 5 times (from 24 to 128). The observed increase was confined mainly to the Northern and Central regions, while no increase was observed in the South of the country.

## Discussion

This is the first population-based survey performed at a national level exploring the places of care, the provision of and the access to palliative care services of Italian cancer patients in their last three months of life. The validity and generalizability of these results must be interpreted taking into account strengths and limitations of the study design [21]. Although the post-bereavement surveys overcome problems related with prospective studies impaired by the practical difficulties of obtaining representative cohorts of terminal cancer patients [24], evaluating to what extent the information regarding the access and utilization of palliative care services is valid and accurate may be problematic. In this survey, only information on the domiciliary referral of patients for PCTs (and the date of their first visit) was obtained from the original case sheets. Conversely, information on the palliative care support received in hospital, as socio demographic and clinical characteristics of the patients and their caregiver, was collected through interviews with the bereaved non-professional caregivers.

A review of studies that compares patients' and proxies' view [25] suggests that proxies can reliably report on the quality of services and objective symptoms. For pain, anxiety and depression, that are more subjective aspects of the patient's experience, the agreement is poorest. Our findings are supported by the validity of the information reported by the caregivers regarding patients' referral to domiciliary PCTs. These results emphasize the appropriateness of the post-bereavement surveys as a methodology to gather valid data on practical aspects of the patient's experience, and support the use of the caregivers as a source of valid information [25].

The distribution of care settings throughout country was polarised into two main places: home and hospital – where most cancer patients spend the entire or part of their last three months of life. Significant geographical differences were observed in the combination of these places of care. Similar differences were observed as regards the place of death of cancer patients in Italy [26] and within Italian regions [27]. The observed broad differences in place of death and of care across the country, suggest an inappropriate use of hospital resources during the terminal phase of disease.

A very low proportion of Italian cancer patients received palliative care support: 14% of those cared for at home and 20% of those admitted to hospital.

Population-based palliative care studies recently carried out in different countries, showed a coverage proportion ranging between 19% and 68% [9,10,12,28,29].

Italian cancer patients received palliative care at home, with clear geographical differences reflecting an unequal provision of and access to palliative care services across the country. Only a few districts in the South of Italy offer PCTs with limited access. Conversely, the similar proportion of patients receiving palliative care in the other regions of the country, is the product of an unequal combination of different provision of services (higher in the North West) and of referrals to the existing services (higher in the North East and in the Centre). The analysis of the trend over time in terms of provision of services shows an unchanged availability of domiciliary PCTs and a significant increase in the provision of hospices. Unfortunately, this change, limited to the North and, to a lesser extent, to the Centre of Italy, worsens the overall situation in terms of equity. The increased availability of hospices is largely due to the policy of the Ministry of Health, strongly supporting and funding this type of service [30], rather than funding a global palliative care program. Moreover, in the absence of any national instrument to promote, support, and assess the development of the program, and with a federal government of the Health Service, the more developed regions of the North of Italy are more likely to make use of national funds to create or expand palliative care services. As a consequence, inequities are expected to become even more marked in the near future.

Geographic variation in the availability and provision of palliative care programs, is present in others countries where there has been a rapid and unplanned development of services and programs [4-6,28].

For Italian cancer patients admitted to hospital, no significant difference was found between the four geographical areas and the admission wards. An Italian survey carried out in 40 hospitals aimed at evaluating how people die in hospital general wards, showed that acute inpatient institutions in Italy are inadequate to cope with the needs of dying patients [19]. Independently from the geographical areas, hospital PCTs in Italian hospitals are lacking, and even pain counselling from a is available for only a minority of patients.

According with the literature [4,7], Italian cancer patients cared for at home and referred for domiciliary PCTs, differ significantly from not referred patients for a number of factors. The multivariate logistic regression showed that the most important determinants of referral are the time window from diagnosis to death and the caregivers' educational level. Generally, the conditions of short-term survivors can rapidly alter, and the dying trajectory unpredictable [12]. For this group of patients it is more difficult to establish links with community support, to plan and organize the referral for domiciliary PCTs [7,8,13]. The caregiver's educational level, often an index of their socio-economic status, affects the skill and ability to identify and obtain the more appropriate healthcare services [7,31].

The multivariate logistic regression analysis also revealed that the Italian cancer patients who are elderly and supported by a caregiver with a low educational level are less likely to be referred to a pain specialist whilst in hospital. A number of reasons for non referral of older people to palliative care have been reported in medical literature suggesting that older patients might experience less pain, less-severe symptoms and less psychological distress than younger patients [4,7,11,12]. This may be reflected in an obsolete view of some physicians involved in cancer care and research, who do not consider adapting care to the needs of patients of different age groups [9]. Moreover, there is evidence that pain is underreported and under treated among elderly cancer patients [32]. The considerations reported for palliative home care services regarding the educational level of the caregiver, can be also be applied in this context.

## Conclusion

The results of this survey show that both the provision of palliative care services and the access to existing services, are inadequate in quantity and, more important, unequally distributed across Italy. Moreover, the analysis of determinants of referral identifies the level of education of both the patients and the caregivers as an important factor of access to the health services. It is important to note that inequity in provision and access puts patients and families who are already socially disadvantaged at a further disadvantage with respect to their health [33]. This may result from a spontaneous and unregulated development of palliative care services without any national program for the assessment of the quality of care provided. In the absence of a specific plan targeted at reducing inequalities, the simple allocation of funds for the development of new palliative care services might even worsen the situation. As from 2006 palliative care for cancer patients must be provided by the National Health System, and health policy-makers should invest in funding effective, palliative care services and networks both at home and in hospital. A monitoring system aimed at identifying inequities both in provision and in access is needed to ensure equitable access to palliative care for all members of society.

## Competing interests

The author(s) declare that they have no competing interests.

## Authors' contributions

MC is the principal investigator of the survey and guarantor. MC and MB designed the survey and the research materials. All the members of the ISDOC study group (Appendix 1) discussed and approved the final protocol of the survey. MC and MB coordinated the survey at a national level. MB, MC and DFM analysed the data, interpreted and discussed the results presented in this article. This paper was primarily written by MB and MC, and then revised, discussed and emended by all the authors that approved the final version of the manuscript.

## Appendix 1

### The ISDOC study group

**Massimo Costantini, Monica Beccaro, Maria Pia Sormani, Paolo Bruzzi **(Unit of Clinical Epidemiology, National Cancer Institute, Genova); **Domenico Franco Merlo **(Unit of Epidemiology and Biostatistics, National Cancer Institute, Genoa); **Gabriella Morasso, Silvia Di Leo **(Psychology Service, National Cancer Institute, Genoa); **Paolo Giorgi Rossi, Piero Borgia **(Agency for Public Health, Lazio Region, Rome); **Maurizio Montella, Maria Grimaldi **(Department of Epidemiology, National Cancer Institute, G.Pascale Foundation, Naples); **Eugenio Paci, Nicoletta Susini, Riccardo Cecioni, Guido Miccinesi **(Clinical Epidemiology, Centre for the Study and Prevention of Cancer, Florence); **Renato Pisanti **(Labos Foundation, Rome).

## Pre-publication history

The pre-publication history for this paper can be accessed here:


